# FDA and EMA approval of datopotamab-deruxtecan: A paradigm shift in breast cancer drug evaluation?

**DOI:** 10.1016/j.breast.2025.104589

**Published:** 2025-09-23

**Authors:** Armando Orlandi

**Affiliations:** Comprehensive Cancer Center, UOC Medical Oncology, Fondazione Policlinico Universitario Agostino Gemelli IRCCS, Roma, Italy

## Abstract

The January 2025 FDA and EMA approvals of datopotamab-deruxtecan for metastatic HR-positive, HER2-negative breast cancer represents an unprecedented regulatory decision. The TROPION-Breast01 trial demonstrated significant progression-free survival improvement (6.9 vs 4.9 months; HR 0.63) but failed to show overall survival benefit (18.6 vs 18.3 months; HR 1.01)[1,2]. This marks the first full approval of a breast cancer therapeutic that failed a co-primary overall survival endpoint. Subsequently, the European Medicines Agency also granted marketing authorization in January 2025, creating a concerning precedent across both major regulatory agencies. With both major regulatory agencies now having approved this agent, breast cancer specialists must grapple with fundamental questions: What constitutes meaningful clinical benefit in heavily pretreated patients? How should we interpret trials with divergent endpoint results? This commentary examines the implications for breast cancer practice and argues for urgent reassessment of evidence standards as the therapeutic landscape evolves.

## 1 Introduction

The synchronized approvals by both FDA and EMA of datopotamab-deruxtecan for metastatic HR-positive, HER2-negative breast cancer has created unprecedented challenges for breast cancer specialists. For the first time in modern oncology, both major regulatory agencies have granted full approval to a breast cancer drug that failed to demonstrate survival benefit in a trial specifically designed with dual co-primary endpoints, representing a fundamental shift in global regulatory standards that demands critical examination.

The growing disconnect between progression-free survival and overall survival benefits in cancer therapeutics has been extensively documented across multiple tumor types [3,4], yet the regulatory response to this phenomenon remains inconsistent and concerning.

This decision forces us to confront fundamental questions about evidence standards in breast cancer therapeutics. As clinicians treating patients with advanced disease, how do we counsel patients about a treatment that clearly delays progression but does not extend life? What does this approval mean for the future of clinical trial design and regulatory standards in our field? Most importantly, how do we maintain scientific rigor while ensuring patient access to potentially beneficial therapies?

## 2 The TROPION-Breast01 Dilemma

The TROPION-Breast01 trial enrolled 732 patients with metastatic HR-positive, HER2-negative breast cancer who had progressed on endocrine therapy and received at least one prior chemotherapy regimen [1]. The study design followed established principles with appropriate statistical power for both progression-free survival and overall survival as co-primary endpoints.

The results present a statistical paradox that challenges our understanding of drug efficacy in breast cancer. The robust 37 % reduction in progression risk (HR 0.63, 95 % CI: 0.52–0.76) should theoretically translate to some survival benefit, yet the overall survival hazard ratio was 1.01—indicating no benefit whatsoever [2].

This disconnect is particularly striking given historical data in breast cancer showing strong correlations between progression-free survival and overall survival improvements [5,6]. The completely neutral overall survival result, with post-progression survival was similar between arms (11.7 vs 13.4 months), raises fundamental questions about the clinical meaningfulness of the observed progression-free survival benefit and challenges the premise that delaying progression necessarily translates to patient benefit.

### 2.1 Clinical practice implications

#### 2.1.1 Patient communication challenges

This approval creates unprecedented challenges for patient counseling. When patients with heavily pretreated metastatic breast cancer ask, "Will this treatment help me live longer?"—perhaps the most fundamental question in oncology—we must now answer based on evidence showing clear progression delay but no survival benefit.

The ethical implications of recommending treatments with proven progression-free survival benefit but no survival advantage have been extensively debated in the oncology literature [7,8], yet regulatory approvals continue to outpace our professional consensus on appropriate evidence thresholds.

The superior tolerability profile of datopotamab-deruxtecan compared to chemotherapy (20.8 % vs 44.7 % Grade 3+ adverse events) represents genuine clinical value. However, this must be balanced against the substantial cost—typically $150,000-$200,000 annually for antibody-drug conjugates—without demonstrated survival benefit.

#### 2.1.2 Treatment sequencing considerations

The approval complicates treatment sequencing decisions in metastatic HR-positive breast cancer. We now have three antibody-drug conjugates available: sacituzumab-govitecan, trastuzumab-deruxtecan (for HER2-low disease), and datopotamab-deruxtecan. Unlike its counterparts, datopotamab-deruxtecan lacks survival data, yet carries full regulatory approval.

How should we sequence these therapies? Should we prioritize treatments with survival benefit over those with progression-free survival benefit alone? These questions have immediate practical implications for daily clinical practice.

#### 2.1.3 Regulatory harmonization and its consequences

The European Medicines Agency's January 2025 approval decision represents a concerning harmonization of regulatory standards around progression-free survival endpoints. This synchronized approach across major regulatory bodies eliminates the potential for divergent evidence evaluation that might have provided valuable insights into optimal approval standards. The alignment of FDA and EMA decisions, while facilitating global drug access, raises questions about independent regulatory evaluation and the potential for regulatory capture by industry standards that prioritize speed to market over evidence quality.

### 2.2 Broader implications for breast cancer research

#### 2.2.1 Trial design considerations

This approval may influence how pharmaceutical companies design future breast cancer trials. If progression-free survival alone becomes sufficient for full approval, we might see:

• Shorter follow-up periods inadequate for meaningful survival assessment
• Reduced investment in combination strategies that might provide survival benefit
• Increased focus on surrogate endpoints over patient-centered outcomes

Similar concerns have been raised regarding accelerated approvals in early breast cancer, where event-free survival benefits have not consistently translated to overall survival improvements [9,10], suggesting this regulatory pattern extends beyond the metastatic setting.

#### 2.2.2 Evidence standards evolution

The breast cancer community must consider whether our evidence standards should evolve with the therapeutic landscape. The rapidly changing treatment environment during TROPION-Breast01—with multiple new therapies becoming available—may have confounded survival results through post-progression treatment effects.

However, accepting progression-free survival as sufficient for approval without survival benefit sets a concerning precedent.

This precedent extends beyond breast cancer, as similar regulatory patterns have emerged in lung cancer, gastrointestinal malignancies, and hematologic cancers, suggesting a broader erosion of evidence standards across oncology [11,12].

### 2.3 The path forward

#### 2.3.1 Clinical practice: A more focused approach

Given the regulatory precedent now established, clinical practice must adapt through enhanced informed consent processes that clearly distinguish between progression delay and survival benefit, evidence-based treatment sequencing that prioritizes survival-proven therapies when available, and robust real-world evidence generation to identify potential patient subgroups who might derive survival benefit.

#### 2.3.2 Regulatory considerations

The breast cancer community should advocate for:

• Clear guidelines on co-primary endpoint interpretation
• Mandatory post-marketing requirements for extended survival follow-up
• Transparency in regulatory decision-making processes
• Continued emphasis on patient-centered outcomes

#### 2.3.3 A call for dialogue

This approval represents more than a single regulatory decision—it reflects evolving approaches to evidence evaluation in breast cancer therapeutics. Rather than simply accepting or rejecting this precedent, the breast cancer community should engage in thoughtful dialogue about optimal evidence standards.

We must balance several competing priorities: providing access to potentially beneficial therapies, maintaining rigorous evidence standards, optimizing healthcare resource allocation, and preserving patient trust in our therapeutic recommendations.

The precedent established by the datopotamab-deruxtecan approval demands immediate professional society guidance and potentially formal position statements from major oncology organizations regarding acceptable evidence thresholds for regulatory approval.

## 3 Conclusion

The datopotamab-deruxtecan approval challenges fundamental assumptions about evidence standards in breast cancer therapeutics. While the drug clearly provides clinical benefit through progression delay and improved tolerability, the absence of survival improvement combined with regulatory approval across major agencies represents a fundamental shift in evidence evaluation that demands critical examination from the breast cancer community.

As breast cancer specialists, we must thoughtfully navigate this new landscape, ensuring that our treatment recommendations remain grounded in evidence while acknowledging the complexities of modern drug development. The synchronized regulatory approach across major agencies eliminates the opportunity for comparative evidence evaluation and demands that the medical community take leadership in defining appropriate evidence standards.

Ultimately, our patients deserve therapies that provide meaningful clinical benefit. Whether progression delay without survival improvement meets this standard in heavily pretreated metastatic breast cancer remains an open question that merits continued discussion within our community.

## Funding

No specific funding was received for this work.

## Declaration of competing interest

AO received fee for speaking from: AstraZeneca, Daiichi Sankyo, Novartis, Lilly, Pfizer, AbbVie, Gilead.

## References

[1] Bardia A., Jhaveri K., Kalinsky K. (2024). Datopotamab deruxtecan versus chemotherapy in previously treated inoperable/metastatic hormone receptor–positive human epidermal growth factor receptor 2–negative breast cancer: primary results from TROPION-Breast01. J Clin Oncol.

[2] AstraZeneca (September 2024).

[3] Burzykowski T., Molenberghs G., Buyse M. (2001). Validation of surrogate end points in multiple randomized clinical trials with failure time end points. J R Stat Soc Ser C Appl Stat.

[4] Shi Q., Sargent D.J. (2009). Meta-analysis for the evaluation of surrogate endpoints in cancer clinical trials. Int J Clin Oncol.

[5] Michiels S., Pugliano L., Marguet S. (2016). Progression-free survival as surrogate end point for overall survival in clinical trials of HER2-targeted agents in HER2-positive metastatic breast cancer. Ann Oncol.

[6] Beaver J.A., Howie L.J., Pelosof L. (2018 Jun 1). A 25-year experience of US food and drug administration accelerated approval of malignant hematology and oncology drugs and biologics: a review. JAMA Oncol.

[7] Yoon N.R., Na Y.J., Lee J.H., Song I., Lee E.K., Park M.H. (2024 Mar 4). Evaluation of changes in the clinical benefits of oncology drugs over time following reimbursement using the ASCO-VF and the ESMO-MCBS. J Cancer Res Clin Oncol.

[8] Saltz L. (2016). Perspectives on cost and value in cancer care. JAMA Oncol.

[9] Prat A., Pascual T., De Angelis C. (2020). HER2-enriched subtype and ERBB2 expression in HER2-positive breast cancer treated with dual HER2 blockade. J Natl Cancer Inst.

[10] Sparano J.A., Gray R.J., Ravdin P.M. (2019). Clinical and genomic risk to guide the use of adjuvant therapy for breast cancer. N Engl J Med.

[11] Sridhara R., Johnson J.R., Justice R., Keegan P., Chakravarty A., Pazdur R. (2010). Review of oncology and hematology drug product approvals at the US food and drug administration between July 2005 and December 2007. J Natl Cancer Inst.

[12] Chen E.Y., Joshi S.K., Tran A., Prasad V. (2019). Estimation of study time reduction using surrogate end points rather than overall survival in oncology clinical trials. JAMA Intern Med.

